# Seasonality and Dynamic Spatial Contagion of Air Pollution in 42 Chinese Cities

**DOI:** 10.1155/2013/381410

**Published:** 2013-03-03

**Authors:** Zhanqiong He, Songsak Sriboonchita, Min He

**Affiliations:** ^1^Faculty of Management and Economics, Lianhua Campus, Kunming University of Science and Technology, Kunming 650093, China; ^2^Faculty of Economics, Chiang Mai University, Chiang Mai 50200, Thailand; ^3^Faculty of Transportation Engineering, Chenggong Campus, Kunming University of Science and Technology, Kunming 650504, China

## Abstract

To monitor and improve the urban air quality, the Chinese government has begun to make many efforts, and the interregional cooperation to cut and improve air quality has been required. In this paper, we focus on the seasonality of the first and second moments of the daily air pollution indexes (APIs) of 42 Chinese sample cities over 10 years, from June 5, 2000 to March 4, 2010, and investigate the dynamic correlation of air pollution indexes (APIs) between 42 Chinese cities and their corresponding regional and national levels; comparison with the model without seasonal consideration is made. By adopting a DCC-GARCH model that accounts for the seasonality, we found that (i) the transformed DCC-GARCH model including seasonality dummies improves the estimation result in this study; (ii) the seasonality feature of the second moment follows that of the first moment, with the condition mean and variance of the second and autumn significantly lower than spring, whereas that of winter is higher than spring; (iii) the correlation between local APIs and their corresponding regional and national levels is dynamic; (iv) comparing with the DCC-GARCH model estimation, the transformed model does not change the feature of the dynamic correlations very much.

## 1. Introduction

With the increasing concern of environmental problem and public health, Chinese government began to struggle the urban air quality problem. Many efforts have been made by the national and local authorities. Some cities began to restrict the car ownership; policies encouraging low-carbon economy are established and implemented. Moreover, the interregional cooperation to cut and improve air quality has been required. The three key regions required to carry out the interregional cooperation are in northern China which includes Beijing, Tianjin, and Hebei; Yangtze River Delta; Pearl River Delta. The three regions are the most developed and populated ones. Their cooperation seems more urgent, and their cooperation will provide experiences for other, regional kinds of and national cooperation. As a big country with wide territory, investigating more sample cities is necessary, since it will provide more information for further research and policy decision.

Air quality is a public good that generates transboundary spillovers. Cooperation between cities, regions, or even countries is very important. Good understanding of spatial contagion is very important for the air pollution control cooperation. So, it is important to study the dynamic correlations between local, regional, and national levels, by then providing support for intraregional, interregion, and national cooperation.

Some previous studies have surveyed the regional nature of the air pollution. Miller and Grumet [1] provide an overview of the scientific basis that supports the actions of the USA and Canada to implement regional ozone pollution control measures. Cousin et al. [2] studied the synoptic and local meteorological situation in the Marseille region, Europe, and found that ozone levels are due to both regional and local factors. By using a time series, cross-sectional panel of pollution data from Hong Kong and southern China, along with weather variables from Hong Kong and employing an econometrically-based structural model, Xiao et al. [3] conducted Granger causality tests and found that pollutants PM10 and NO_2_ causality runs in both directions, which confirmed the regional nature of the air pollution problem.

More studies focus on the impact of emission or dust from one region to another. Zhang et al. [4] examined the impact of tripled anthropogenic emissions from China and India over the base level (gaseous species and carbonaceous aerosols for 2000) on air quality over the USA. The simulations indicate an extensive area of elevated pollutant concentrations spanning from the Arabian Sea to the Northern Pacific and to the northern Atlantic. Holloway et al. [5] studied the geographic variability and seasonality of the inflow of global pollutants to Asia. They found that imported O_3_ contributes significantly throughout Asia. In detail, both North America and Europe contribute to ground-level O_3_  concentrations throughout the region, though the seasonality of these two sources varies. Based on aluminium and calcium concentrations in PM10, Lee et al. [6] studied the transport of dusts from east Asian and noneast Asian sources to Hong Kong and found that dust events (96%) involve noneast Asian sources. Liu et al. [7] studied the influence of Asian dust storms on air quality in Taiwan. The high PM10 concentration on the east coast is associated with long-range transported dust, but the high PM10 concentration at the western side is due to a mixture of dust particles and local pollutants.

Seasonality is another focus of former researches. Zhang et al. [8] found that total suspended particle (TSP) was the chief pollutant influencing air pollution index (API) in northern China in spring and winter seasons. Sand and dust storm might be a major factor affecting the temporal variability and spatial distribution of TSP and dust fall in China. Grivas et al. [9] analyzed PM10 concentration data collected at 8 sites over the Greater Athens. Statistical examination showed that the seasonal variation of PM10 levels was not found to be uniform across the 8 sites, and significant intersite correlations were observed. Yamaji et al. [10] analyzed the seasonal variation of surface O_3_ over Japan. They concluded that surface O_3_ distribution over East Asia varies dynamically from season to season according to the meteorological condition, with O_3_ concentrations characterized by two peaks in spring and autumn and summer minimum. Fang and Chang [11] analyzed atmospheric particulate (PM10 and PM2.5) mass concentration using hourly data collected from seven air quality monitoring stations in Taiwan and found that the highest mean PM2.5 particle concentration was detected during spring, whereas the highest mean PM2.5 particle concentration was recorded during winter. By selecting 4 sites covering urban, suburban, rural, and coastal areas as representatives for detailed analysis, Zheng et al. [12] analyzed Pearl River Delta (PRD) regional air quality monitoring network and investigated the characteristics of ground-level ozone in the region. Their results showed that there are distinct seasonal and diurnal cycles in ground-level ozone across the PRD region. Chan [13] investigated the seasonal variation of different types of particulates in a fixed roadside station in heavily trafficked urban area of Hong Kong. They found that large-size particles had an apparent seasonal variation, with higher concentration level in winter and lower in summer. The dry continental winter monsoon and the wet oceanic summer monsoon are the dominating factors. On the other hand, the variation of PM2.5 is much smaller since they are more affected by local traffic emission. So et al. [14] studied long-term trend and spatial variations of PM2.5 mass and chemical composition in Hong Kong covering 3 sites with different land-use characteristics, namely, roadside, urban, and rural environments. They found that seasonal variations of PM2.5 mass concentration at the 3 sites were similar higher in autumn/winter and lower in summer. Hoti et al. [15] analyzed the trends and volatility in atmospheric carbon dioxide concentration levels using daily data from January 1 1991 to December 31 2002 collected at Ryori, Japan, and Mauna Loa, USA. Their results implied that the two regions, namely, Asia and the southwest Pacific, were independent in terms of the shocks to the ACDC levels. Using hourly data of PM10 concentration, Yang [16] evaluated the spatial and seasonal variations in the four regions in Taiwan, and they found that spatial and seasonal variations of PM10 concentrations are rather large over Taiwan; seasonal variation is characterized by high concentrations in winter and low in summer. Gupta et al. [17] investigated the air quality of urban region of Kolkata, India, consisting of residential, commercial, and industrial sites having high population density and pollution. Winter concentrations of ambient SO_2_, NO_2_, NH_3_, and PM10 were observed to be higher irrespective of the monitoring sites.

The past literature revealed that most studies focusing on the relationship between regions have focused on the impact of emission or dust from one region to another, and mostly between broader regions like countries. Seldom was studied between regions within China, and, in the related research, the integrated APIs were not studied. Up to our knowledge, seasonal variation was studied widely but was mostly focused on the level. Following our previous research [18], by introducing seasonality dummy in the DCC-GARCH model which was an econometric model widely used by many researchers in time-varying correlations finance research, this paper attempts to investigate whether the seasonal effect exists in both first and second moments and how the introduction of seasonal effect affects the dynamic correlations of data series.

This study contributes to the existing literature not only by covering 42 cities of nine regions in China but also by examining both the seasonality of first and second moment and examining their impact on the dynamic correlation between local, regional, and national levels.

The rest of the paper is set up in the following manner.  Section 2 presents the econometric model.  Section 3 contains the description of the data. The empirical results are in Section 4, followed by conclusion in the last section.

## 2. Model

Time-varying correlations are often estimated with multivariate GARCH models. Since Bollerslev et al. [19] originally proposed the multivariate GARCH model in the half-vec (vech) form, many others have been developed, but most are linear in squares and cross products of the data [20]. Engle [20] proposed a dynamic conditional correlation (DCC) models which can be estimated very simply with univariate or two-step methods based on the likelihood function. In this paper, we employed the DCC-GARCH model, after Engle [20], to examine the existence of volatility in each series and the dynamic correlations between urban APIs, regional APIs, and national APIs.

Let us consider the APIs *Y*_*t*_ = (*Y*_1*t*_,…,*Y*_*kt*_)′, for  *t* = 1,…, *T*. The following mean equation was estimated for each series given as

(1) $$\begin{array}{c} Y_{it}=\mu_{i}+aY_{it-1}+\varepsilon_{it}\;\varepsilon_{it}∼N\left(0,H_{t}\right), \end{array}$$

where *Y*_*it*_ is API in series *i* at time *t*, and *ε*_*it*_ is the error term for the API *i* at time *t*. Equation (1) was then tested for the existence of ARCH. All estimated series exhibited evidence of ARCH effects. The DCC parameterization of conditional covariance metrics is given as [20]

(2) $$\begin{array}{c} H_{t}\equiv D_{t}R_{t}D_{t}, \end{array}$$

where *D*_*t*_ is the *k* × *k* diagonal matrix of time-varying standard deviations from univariate GARCH models with ${\sqrt{h}}_{it}$ on the *i*th diagonal, and *R*_*t*_ is the time-varying correlation matrix.

The elements of *D*_*t*_ can be expressed for the univariate form as follows:

(3) $$\begin{array}{c} h_{it}=\omega_{i}+\sum_{p=1}^{p_{i}}a_{ip}r_{it-p}^{2}+\sum_{q=1}^{Q_{i}}\beta_{iq}h_{it-q}, \end{array}$$

for *i* = 1; 2,…*k* with the usual GARCH restrictions for nonnegativity and stationarity being imposed, such as nonnegativity of variances and ∑_*p*=1_^*p*_*i*_^ *a*_*ip*_ + ∑_*q*=1_^*Q*_*i*_^ *β*_*iq*_ < 1.

The proposed dynamic correlation structure is

(4) Qt=(1−∑m=1Mθ1m−∑n=1Nθ2n)Q−+∑m=1Mθ1m(εt−mεt−m′)+∑n=1Nθ2nQt−n,

(5) $$\begin{array}{c} R_{t}=Q_{t}^{*-1}Q_{t}Q_{t}^{*-1}, \end{array}$$

where $\overset{-}{Q}$ is the unconditional covariance of the standardized residuals resulting from the first-stage estimation and

(6) $$\begin{array}{c} Q_{t}^{*}=\left[\begin{array}{ccc} \sqrt{q_{11}} & \cdots & 0\\ \vdots & \ddots & \vdots \\ 0 & \cdots & \sqrt{q_{kk}} \end{array}\right], \end{array}$$

so that *Q*_t_*  is a diagonal matrix composed of the square root of the diagonal elements of *Q*_*t*_. The typical element of *R*_*t*_ will be of the form $\rho_{ijt}=q_{ijt}/\sqrt{q_{ii}q_{jj}}$.

To investigate the seasonal effect of mean and variance and the effect on the dynamic correlation between local APIs, regional APIs, and national APIs, we set three seasonality dummies in both mean and variance equations, so that (1) now becomes

(7) $$\begin{array}{c} Y_{it}=\mu_{i}+S_{2}D_{2}+S_{3}D_{3}+S_{4}D_{4}+aY_{it-1}+\varepsilon_{it}. \end{array}$$

Equation (2) becomes

(8) $$\begin{array}{c} h_{it}=\omega_{i}+S_{2}^{\prime }D_{2}+S_{3}^{\prime }D_{3}+S_{4}^{\prime }D_{4}+\sum_{p=1}^{p_{i}}a_{ip}r_{it-p}^{2}+\sum_{q=1}^{Q_{i}}\beta_{iq}h_{it-q}, \end{array}$$

so that *D* is the seasonal effect vector where *D*_2_, *D*_3_, and *D*_4_ equal 1, when *t* is in summer, autumn, or winter, respectively, and other equations are the same. Spring includes March, April, and May; summer includes June, July, and August; autumn includes September, October, and November; winter includes December, January, and February.

## 3. Data Description

The data series for this study comprises of 42 groups of daily average air pollution indexes (APIs) of 42 Chinese sample cities. Each group consists of three series: city API, their corresponding regional level, and national API during the period from June 5, 2000 to March 4, 2010, that is, we have 3560 observations for each series.

Data on urban air pollution index come from the data base of the Ministry of Environmental Protection of China (http://www.zhb.gov.cn/) (MEPPRC). The reason why we choose the 42 sample cities is that even though the APIs of more cities were reported later on, say 87 cities till June, 2011, the data of these 42 cities were available since June 5, 2000, so we have more observation; moreover, the 42 cities were scattered in the nine regions of China (shown in Table 1). Based on the climate type, regional geographical features, and so forth, there are many ways of Chinese regional divisions. According to Zhen Du et al. [21], Lin Chao, Luo Kaifu, Huang Bingwei, Ren Meie, Zhao Xueyu, Zhao Songqiao, Xi Chengfan, and so forth suggested different schemes. We employed the scheme which is based on the division of Ren Meie and Yang Renzhang.

The data of regional and national levels are integrated from APIs of the other cities within the region and nation, respectively, by calculating inverse distance weighted the average of city APIs for all the other cities in the region and in the nation.

**Figure 1:**
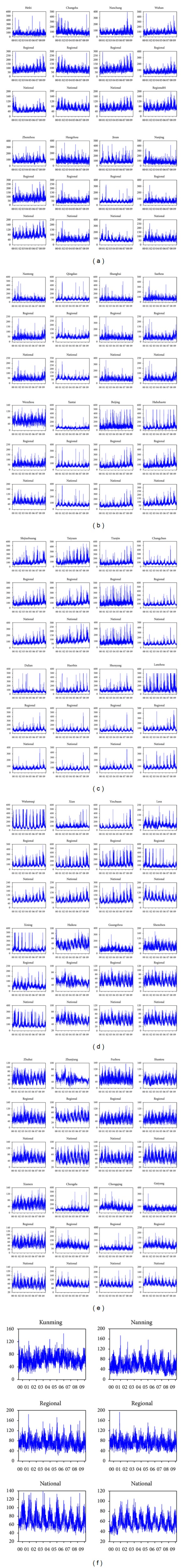
APIs for 42 sample cities and corresponding regional and national levels.

Figure 1 presents the plot of APIs for 42 sample cities and corresponding regional and national levels. These plots are similar in all 126 series, obvious volatility clustering feature can be noticed. Even though, except for few series, seasonal variation of raw data can be noticed, unit root tests exhibit that only the ADF unit root tests without constant and trend of few series are not statistically significant; ADF test of most series and PP test show that all the series are statistically significant, rejecting the hypothesis that there exist unit roots.

## 4. Results

Table 2 reports the estimation result of the two-step DCC-GARCH model based on the univariate GARCH (1, 1) for each series. All the estimates in this paper are obtained using the OxMetrics 5.10 econometric software package developed by Jurgen A. Doornik. The error student distribution assumption has been used in all cases.

In the mean equations, the *c* and AR estimations are all significant at 1% level; except 17 out of 126 series, the ARCH(*α*) estimations are significant at 5% level. Except 7 out of 126 series, the GARCH(*β*) estimation is significant at least at 5% level. Except for the *θ*_1_ estimations of Beijing and Taiyuan, the estimations of DCC parameters, *θ*_1_ and *θ*_2_, are statistically significant at 5% level in all left 40 cases. This indicates that the assumption of constant conditional correlation for all shocks to return is not supported empirically. Except for Tianjin, the short-run persistence of shocks on the dynamic conditional correlations is low, under 0.1 generally, while the long-run persistence of shocks to the conditional correlations is high for most sample cities.

As shown in Table 3, from the results of the tests, the model with seasonality dummies outperform the one without seasonality dummies according to all three criteria, say the Log(L), AIC, and BC.

Table 4 reports the seasonality dummies significance of mean equations and variance equations. The result exhibits both general rules and regional heterogeneity. In general, the seasonality feature of the second moment follows that of the first moment, specifically, if summer is significantly lower than spring in the mean equation, the situation is mostly the same in the variance equation, but, except southern China, when the conditional mean in winter is significantly higher than spring, the variance equation does not follow the same feature. In almost all cities and regions, the condition mean and variance of summer are significantly lower than spring. In several regions, the condition mean and variance of winter are significantly higher than spring.

Focusing on the regional level, Central China, northern China, northwest, northeast, southeast and Southwest exhibit same features, both the conditional mean and the conditional variance of the second and autumn are lower than that of spring, whereas winter is higher than spring; for the Qinghai-Tibet and southern China, both the conditional mean and the conditional variance of the second and autumn are lower than that of spring, where as winter is not significantly different than spring; southern China, which is composed of three cities in Fujian province, is a special one, with both the conditional mean and the conditional variance of summer lower than that of spring, and the third and fourth seasons are higher than spring.

**Figure 2:**
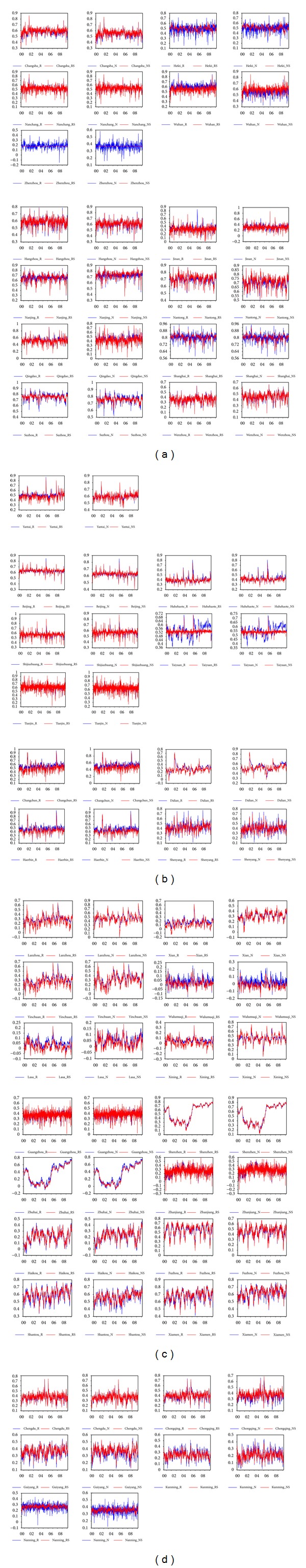
Dynamic correlation comparison.

Figure 2 presents the time-varying conditional correlations between sample cities and their corresponding regional and national levels. The blue lines are the dynamic correlations calculated from DCC-GARCH models without seasonality dummies, while the red lines are from the ones with seasonality dummies. It is clear that there is significant variation in the conditional correlations over time; most sample cities exhibit similar dynamic correlations with what is calculated from DCC-GARCH models without seasonality dummies (blue lines). Northwest and Qinghai-Tibet exhibit low-average relation with regional and national APIs, while East China, North China, and southeast exhibit high relation. Wulumuqi and Lasa exhibit very low relation with regional and national APIs; Xining shows a high relation with national but a low relation with regional API, indicating that cities in vast territory and special terrain are not very sensitive to shocks of regional and national APIs. Several cities exhibit special characters. Zhenzhou has almost zero short-run persistence of shocks to the conditional correlations (Θ_1_) in model with seasonality dummies and exhibits constant correlation accordingly. For Taiyuan, including seasonality dummies decreases the variance of dynamic correlations. Shenzhen and Zhuhai, the two cities with the shortest distance between each other, exhibit a decrease of dynamic correlation with both regional and national levels after spring 2001 and then an increase again after autumn 2004 and reach a relative stable level in summer 2006. Southeast and Qinghai-Tibet exhibit obvious seasonal variation.

## 5. Discussion

Understanding how the mean and variance of the APIs change across season and whether there exists the regional homogeneity concerning seasonality is important in understanding the features of the city APIs and the regional nature of APIs. Tracing the dynamic correlations of city APIs and regional/ national levels is a crucial issue for the understanding of the space relationship of APIs and policy implications. Following our previous study, this paper has investigated the seasonality of the first and the second moments of 42 sample Chinese cities scattered in nine regions. Then, dynamic correlations of 42 sample cities' APIs with the regional and national APIs are investigated; comparison with the ones we got from the model without considering the seasonality is made.

The main results can be summarized in the following several aspects. About the model specification, even though some seasonality dummies are not significant, including the seasonality dummies in the mean and variance equations has improved the model in terms of log-likelihood, AIC, and BC criteria. About the seasonality, seasonality feature of the second moment follows that of the first moment. In most cities and regions, the condition mean and variance of the second and autumn are significantly lower than spring, whereas that of winter is higher than spring. That means that the spring and winter exhibit not only the highest mean but also the highest variance of API. Previous studies found that TSP, PM10, PM2.5, surface O_3_,  SO_2_,  NO_2_, and  NH_3_ are observed higher in winter, and our result found that API (integrated from the observation of three pollutant: reparable particulate matter (PM10), SO_2_, and nitrogen dioxide (NO_2_)), an integrated index, also exhibits the same seasonal variation. About the regional features, Qinghai-Tibet, South China, and Southeast China exhibit special features concerning the seasonality effect. Turning to the dynamic correlations, the dynamic correlations calculated from the seasonality dummies are different from that of the model without seasonality dummies, they are time varying, and moreover they exhibit the same features, indicating that including the seasonality dummies in DCC-GARCH models improved the model estimation, while it did not influence the feature of the dynamic correlations very much.

## 6. Conclusions

These results have strong policy implications. When capturing the regional and interregional relationship, seasonal variation should be taken into consideration. Spring and winter exhibit higher volatility, which means higher uncertainty; regional or single city settlement in air pollution control is important but not enough, and interregional cooperation and national decision are important. The cooperation mechanism should be able to respond. the time-varying nature of conditional correlation; regional heterogeneity should be considered in cooperation policy decision; cooperation among regions with higher correlation and similar correlation feature is better.

The limitations of this study are that the data can be improved in the future when more important observations of air quality like PM2.5 are detected and integrated in the API; moreover, the approximation of national API can be improved, and comparison between other possible divisions of region can be made in the future study.

## Figures and Tables

**Table 1:** Cities of study and corresponding regions.

Region	Cities
Central China	Changsha, Hefei, Nanchang, Wuhan, and Zhenzhou
Easernt China	Hangzhou, Jinan, Nanjing, Nantong, Qingdao, Shanghai, Suzhou, Wenzhou, and Yantai
Northern China	Beijing, Huhehaote, Shijiazhuang, Taiyuan, and Tianjin
Northeast	Changchun, Dalian, Haerbin, and Shenyang
Northwest	Lanzhou, Wulumuqi, Xian, and Yinchuan
Qinghai-Tibet	Lasa and Xining
Southern China	Guangzhou, Shenzhen, Zhuhai, Zhanjiang, and Haikou
Southeast	Fuzhou, Shantou, and Xiamen
Southwest	Chengdu, Chongqing, Guiyang, Kunming, and Nanning

**Table:** (a) Central China

API	c	S_2_	S_3_	S_4_	AR	ω	S_2_′	S_3_′	S_4_′	ARCH(*α*)	GARCH(*β*)	Θ_1_	Θ_2_	Q(5)	Q(10)
Changsha, regional, and national															

Changsha	84.18^† ^ (31.90)	−14.46^† ^ (−4.60)	3.30 (0.97)	0.98 (0.26)	0.67^† ^ (33.02)	250.30^† ^ (5.03)	−152.69^† ^ (−3.81)	−59.93 (−1.69)	−10.65 (−0.24)	0.40^† ^ (5.32)	0.37^† ^ (5.18)	0.03^† ^ (4.02)	0.87^† ^ (21.21)	3.59	5.9
Regional	81.50^† ^ (33.47)	−16.22^† ^ (−5.89)	−2.63 (−0.82)	3.85 (1.28)	0.74^† ^ (54.58)	61.63^† ^ (2.93)	−48.50^† ^ (−2.74)	−23.99 (−1.59)	−12.66 (−0.77)	0.13^† ^ (2.07)	0.73^† ^ (7.80)			2.22	4.12
National	69.86^† ^ (38.40)	−10.80^† ^ (−5.15)	−2.98 (−1.33)	5.32^† ^ (2.40)	0.83^† ^ (68.03)	12.27^† ^ (2.51)	−9.84^† ^ (−2.42)	−4.78 (−1.56)	0.50 (0.17)	0.50^† ^ (0.17)	0.80^† ^ (12.13)			14.8^*♯*^	16.35*

Hefei, regional, and national															

Hefei	82.47^† ^ (18.78)	0.08 (0.02)	2.31 (0.47)	−16.87^† ^ (−3.76)	0.60^† ^ (35.11)	353.46^† ^ (3.65)	−51.99 (−0.74)	−7.16 (−0.10)	−218.45^† ^ (−2.89)	0.40^† ^ (2.96)	0.22 (1.55)	0.01^† ^ (4.22)	0.96^† ^ (81.52)	4.16	4.19
Regional	83.48^† ^ (45.30)	2.57 (0.99)	−0.75 (−0.28)	−15.08^† ^ (−7.00)	0.75^† ^ (56.38)	99.23^† ^ (3.96)	−5.93 (−0.29)	−19.48 (−1.00)	−70.01^† ^ (−3.22)	0.19^† ^ (5.74)	0.49^† ^ (6.83)			3.32	4.9
National	49.53^† ^ (45.06)	4.98^† ^ (3.21)	−2.09 (−1.43)	−6.17^† ^ (−4.86)	0.81^† ^ (64.30)	1.83^*♯*^ (2.42)	−0.19 (−0.32)	−1.66^† ^ (−3.90)	−1.49^*♯*^ (2.45)	0.06^*♯*^ (2.35)	0.93^† ^ (30.22)			36.84^† ^	48.18^† ^

Nanchang, regional, and national															

Nanchang	70.03^† ^ (37.56)	−11.22^† ^ (−5.15)	1.35 (0.53)	3.29 (1.32)	0.66^† ^ (45.11)	37.28^*♯*^ (2.49)	−23.34^*♯*^ (−2.40)	−7.13 (−1.08)	−4.36 (−0.63)	0.15^† ^ (3.18)	0.78^† ^ (12.11)	0.001 (0.77)	0.99^† ^ (44.85)	2.73	13.18
Regional	86.18^† ^ (31.18)	−16.05^† ^ (−5.15)	0.56 (0.16)	4.48 (1.33)	0.74^† ^ (55.09)	150.75^*♯*^ (2.55)	−111.46^*♯*^ (−2.30)	−59.29 (−1.50)	−35.52 (−0.92)	0.19^† ^ (3.73)	0.53^† ^ (4.28)			1.05	4.34
National	69.73^† ^ (40.41)	−11.07^† ^ (−5.51)	−2.03 (−0.90)	4.95^*♯*^ (2.28)	0.81^† ^ (63.94)	24.89^*♯*^ (2.35)	−18.87^*♯*^ (−2.28)	−8.98 (−1.61)	−0.06 (−0.01)	0.14^† ^ (3.88)	0.67^† ^ (6.29)			19.65^† ^	25.84^† ^

Wuhan, regional, and national															

Wuhan	91.54^† ^ (29.18)	−21.13^† ^ (−5.96)	−3.86 (−0.97)	7.19* (1.71)	0.68^† ^ (44.34)	84.21 (1.64)	−71.05 (−1.52)	−39.78 (−0.97)	−32.21 (−0.84)	0.03 (0.72)	0.88^† ^ (13.70)	0.04^*♯*^ (2.15)	0.83^† ^ (6.10)	3.19	3.94
Regional	78.82^† ^ (39.56)	−13.82^† ^ (−6.15)	0.71 (0.27)	2.65 (1.06)	0.73^† ^ (52.61)	81.45^† ^ (4.74)	−55.86^† ^ (−4.20)	−19.16* (−1.74)	−5.76 (−0.43)	0.23^† ^ (4.30)	0.55^† ^ (7.64)			5.13	5.81
National	71.22^† ^ (44.53)	−11.65^† ^ (−6.36)	−2.44 (−1.17)	4.33^*♯*^ (2.12)	0.80^† ^ (61.94)	25.73^† ^ (3.91)	−19.0^† ^ (−3.70)	−8.22^*♯*^ (−2.24)	1.66 (0.38)	0.19^† ^ (4.88)	0.62^† ^ (8.69)			21.68^† ^	24.17^† ^

Zhenzhou, regional, and national															

Zhenzhou	81.00^† ^ (36.24)	−11.65^† ^ (−4.77)	−5.29^*♯*^ (−2.08)	4.63* (1.75)	0.63^† ^ (37.22)	59.93 (1.40)	−43.24 (−1.39)	−24.84 (−1.11)	−17.26 (−1.00)	0.11^*♯*^ (2.25)	0.80^† ^ (7.33)	0.001^† ^	0.99^† ^ (2945)	10.01*	17.2469*
Regional	84.81^† ^ (32.85)	−17.75^† ^ (−6.12)	−1.91 (−0.57)	3.25 (1.04)	0.73^† ^ (52.34)	101.93^*♯*^ (2.57)	−78.53^*♯*^ (−2.48)	−41.77* (−1.67)	−27.67 (−1.13)	0.14^† ^ (2.77)	0.68^† ^ (6.03)			1.22	3.34
National	75.82^† ^ (40.75)	−10.62^† ^ (−5.14)	−3.36 (−1.59)	7.16^† ^ (3.00)	0.80^† ^ (59.37)	33.88^† ^ (3.06)	−26.83^† ^ (−2.79)	−16.93^*♯*^ (−2.16)	−0.62 (−0.09)	0.22^† ^ (7.25)	0.58^† ^ (8.15)			5.95	6.72

**Table:** (b) Eastern China

API	c	S_2_	S_3_	S_4_	AR	ω	S_2_′	S_3_′	S_4_′	ARCH (*α*)	GARCH (*β*)	Θ_1_	Θ_2_	Q(5)	Q(10)
Hangzhou, regional, and national															

Hangzhou	84.28^† ^ (43.94)	−16.09^† ^ (−7.02)	−4.34* (−1.65)	1.83 (0.64)	0.59^† ^ (37.87)	252.22^† ^ (3.83)	−142.58^† ^ (−3.43)	−11.29 (−0.33)	100.51 (1.46)	0.16^† ^ (3.03)	0.42^† ^ (3.26)	0.04* (1.92)	0.86^† ^ (6.20)	1.04	1.98
Regional	52.56^† ^ (36.86)	−9.29^† ^ (−5.38)	−5.62^† ^ (−3.15)	−1.46 (−0.74)	0.66^† ^ (43.17)	20.15^*♯*^ (2.47)	−13.36^*♯*^ (−2.44)	−7.04 (−1.58)	1.70 (0.37)	0.13^† ^ (3.81)	0.80^† ^ (14.08)			5.05	7.05
National	57.05^† ^ (39.30)	−10.00^† ^ (−5.83)	−4.64^*♯*^ (−2.56)	0.35 (0.17)	0.70^† ^ (48.77)	16.51^† ^ (2.66)	−11.39^† ^ (−2.60)	−5.43* (−1.64)	1.91 (0.56)	0.13^† ^ (3.80)	0.79^† ^ (14.13)			19.26^† ^	29.89^† ^

Jinan, regional, and national															

Jinan	94.30^† ^ (26.63)	−18.94^† ^ (−5.15)	−13.88^† ^ (−3.45)	6.018 (1.14)	0.65^† ^ (31.47)	441.31^† ^ (4.97)	−327.54^† ^ (−4.45)	−211.02^† ^ (−3.22)	4.36 (0.05)	0.40^† ^ (5.47)	0.25^*♯*^ (2.47)	0.05^† ^ (3.55)	0.74^† ^ (7.56)	1.53	3.25
Regional	61.49^† ^ (34.08)	−11.27^† ^ (−5.49)	−6.51^† ^ (−3.34)	3.90* (1.93)	0.67^† ^ (41.06)	89.43^*♯*^ (2.54)	−74.90^*♯*^ (−2.28)	−68.73^*♯*^ (−2.17)	−55.04* (−1.83)	0.18^† ^ (4.14)	0.60^† ^ (7.67)			8.83	8.88
National	77.89^† ^ (37.26)	−10.10^† ^ (−4.54)	−5.00^*♯*^ (−2.22)	6.59^† ^ (2.94)	0.74^† ^ (51.95)	66.97^† ^ (2.70)	−53.39^*♯*^ (−2.41)	−31.58* (−1.72)	−19.89 (−1.13)	0.29^† ^ (6.03)	0.53^† ^ (6.06)			5.19	6.41

Nanjing, regional, and national															

Nanjing	84.59^† ^ (35.33)	−12.94^† ^ (−4.44)	−4.87* (−1.63)	−2.49 (−0.80)	0.62^† ^ (36.80)	86.66* (1.66)	−56.62* (−1.87)	−21.73 (−1.11)	−15.35 (−0.94)	0.11^*♯*^ (2.29)	0.80^† ^ (7.90)	0.03^† ^ (3.77)	0.93^† ^ (32.25)	9.25*	24.68^† ^
Regional	65.33^† ^ (31.32)	−10.08^† ^ (−4.24)	−4.55* (−1.85)	2.81 (0.94)	0.67^† ^ (42.81)	101.86^† ^ (3.59)	−65.35^† ^ (−3.38)	−38.88^*♯*^ (−2.39)	2.39 (0.16)	0.27^† ^ (3.24)	0.44^† ^ (3.45)			2.09	8.39
National	73.12^† ^ (41.56)	−13.82^† ^ (−6.86)	−5.00^*♯*^ (−2.22)	2.45 (1.02)	0.72^† ^ (51.96)	26.34^† ^ (3.06)	−20.10^† ^ (−3.07)	−8.54* (−1.87)	−0.09 (−0.02)	0.11^† ^ (4.38)	0.80^† ^ (16.07)			9.65*	10.55

Nantong, regional, and national															

Nantong	68.19^† ^ (14.36)	−10.52^*♯*^ (−2.18)	−6.40 (−1.30)	6.38 (1.02)	0.66^† ^ (34.22)	280.73^† ^ (3.17)	−202.64^† ^ (−2.77)	−159.47^*♯*^ (−2.26)	−69.24 (−0.96)	0.34^† ^ (3.24)	0.43^† ^ (2.89)	0.04^† ^ (5.33)	0.91^† ^ (41.58)	2.94	3.54
Regional	49.22^† ^ (37.62)	−8.15^† ^ (−5.11)	−4.11^*♯*^ (−2.43)	−1.50 (−0.79)	0.63^† ^ (38.48)	16.03^† ^ (2.62)	−9.51^*♯*^ (−2.36)	−3.32 (−0.99)	6.10 (1.17)	0.11^† ^ (3.77)	0.83^† ^ (18.27)			7.8	10.84
National	52.24^† ^ (40.25)	−8.73^† ^ (−5.55)	−3.89^*♯*^ (−2.32)	−0.56 (−0.30)	0.65^† ^ (41.65)	15.02^† ^ (2.64)	−9.09^*♯*^ (−2.43)	−3.02 (−1.01)	5.42 (1.22)	0.11^† ^ (3.70)	0.82^† ^ (16.74)			17.74^† ^	27.74^† ^

Qingdao, regional, and national															

Qingdao	80.72^† ^ (30.88)	−19.02^† ^ (−6.53)	−11.33^† ^ (−4.06)	6.48^*♯*^ (2.25)	0.55^† ^ (27.15)	512.10^*♯*^ (2.22)	−445.14^*♯*^ (−2.04)	−422.50^*♯*^ (−1.97)	−373.96* (−1.82)	0.17^† ^ (3.24)	0.47^† ^ (3.63)	0.05^† ^ (4.13)	0.83^† ^ (13.74)	2.42	2.58
Regional	65.33^† ^ (41.43)	−11.50^† ^ (−6.77)	−7.78^† ^ (−4.47)	1.29 (0.69)	0.66^†^ (41.60)	65.79^†^ (3.60)	−50.91^†^ (−3.02)	−40.75^*♯*^ (−2.44)	−26.38 (−1.47)	0.19^†^ (5.91)	0.60^† ^ (6.36)			1.53	2.29
National	70.23^† ^ (36.82)	−10.27^† ^ (−5.29)	−5.43^† ^ (−2.75)	5.43^† ^ (2.61)	0.72^† ^ (46.27)	40.47^† ^ (3.75)	−31.59^† ^ (−3.77)	−23.64^† ^ (−3.33)	−13.54* (−1.71)	0.25^† ^ (7.36)	0.60^† ^ (6.31)			6.23	8.1

Shanghai, regional, and national															

Shanghai	63.75^† ^ (11.52)	−4.78 (−0.88)	−1.40 (−0.25)	10.33 (1.48)	0.58^† ^ (23.54)	611.84^† ^ (4.24)	−474.37^† ^ (−3.36)	−382.55^† ^ (−2.67)	−200.07 (−1.50)	0.43^† ^ (3.45)	0.17 ^*♯*^ (2.14)	0.02^† ^ (3.86)	0.96^† ^ (59.35)	2.97	3.04
Regional	73.45^† ^ (36.31)	−13.11^† ^ (−5.46)	−7.24^† ^ (−2.85)	−0.100 (−0.03)	0.65^† ^ (43.49)	49.66^*♯*^ (2.11)	−34.73^*♯*^ (−2.19)	−19.47* (−1.66)	−0.33 (−0.03)	0.12^† ^ (3.66)	0.80^† ^ (11.40)			12.61^*♯*^	15.55
National	73.46^† ^ (36.57)	−12.99^† ^ (−5.52)	−6.54^† ^ (−2.60)	0.79 (0.28)	0.66^† ^ (45.32)	43.98^*♯*^ (2.06)	−31.01^† ^ (−2.13)	−16.77 (−1.60)	0.15 (0.02)	0.13^† ^ (3.46)	0.79^† ^ (10.58)			19.17^† ^	23.57^† ^

Suzhou, regional, and national															

Suzhou	77.68^† ^ (40.11)	−12.25^† ^ (−5.04)	−6.02^*♯*^ (−2.30)	−3.67 (−1.27)	0.58^† ^ (34.60)	53.20^† ^ (2.60)	−30.28^*♯*^ (−2.43)	−10.57 (−1.01)	16.75 (1.00)	0.11^† ^ (3.58)	0.82^† ^ (16.89)	0.02^*♯*^ (2.01)	0.89^† ^ (12.60)	2.65	6.38
Regional	45.26^† ^ (23.51)	−8.72^† ^ (−4.14)	−5.66^*♯*^ (−2.53)	1.22 (0.46)	0.70^† ^ (42.74)	26.20 (1.24)	−19.78 (−1.29)	−13.00 (−1.10)	−4.10 (−0.59)	0.18 (1.79)	0.74^† ^ (4.44)			1.79	2.2
National	47.20^† ^ (23.76)	−7.65^† ^ (−3.38)	−3.39 (−1.48)	3.14 (1.09)	0.72^† ^ (44.55)	53.02^*♯*^ (2.34)	−37.24^*♯*^ (−2.33)	−24.45* (−1.85)	−2.92 (−0.33)	0.31^† ^ (2.74)	0.46^† ^ (2.60)			9.01	64.59^† ^

Wenzhou, regional, and national															

Wenzhou	69.56^† ^ (56.35)	−13.53^† ^ (−9.05)	−11.44^† ^ (−7.37)	−4.42^*♯*^ (−2.53)	0.61^† ^ (40.12)	120.45^† ^ (4.89)	−67.08^† ^ (−4.24)	−46.75^† ^ (−3.27)	7.38 (0.56)	0.13^† ^ (4.33)	0.34^† ^ (3.07)	0.03^† ^ (3.95)	0.89^† ^ (21.94)	1.36	7.69
Regional	59.83^† ^ (38.33)	−10.31^† ^ (−5.58)	−3.87* (−1.91)	1.70 (0.78)	0.66^† ^ (42.18)	80.09* (1.71)	−51.41* (−1.72)	−24.80 (−1.35)	10.60 (0.74)	0.19^*♯*^ (2.52)	0.52^*♯*^ (2.30)			5.35	15.41
National	64.36^† ^ (41.67)	−10.83^† ^ (−5.98)	−3.94* (−1.94)	3.18 (1.56)	0.77^† ^ (55.84)	24.59 (1.34)	−16.99 (−1.35)	−8.22 (−1.13)	5.36 (1.32)	0.12^*♯*^ (2.31)	0.67^† ^ (3.45)			1.04	4.72

Yantai, regional, and national															

Yantai	65.04^† ^ (32.78)	−10.58^† ^ (−5.04)	−6.79^† ^ (−3.10)	1.20 (0.52)	0.58^† ^ (24.85)	54.92^† ^ (2.32)	−41.20* (−1.92)	−35.50* (−1.68)	−35.29* (−1.66)	0.18^† ^ (3.19)	0.76^† ^ (17.59)	0.02^† ^ (3.50)	0.91^† ^ (27.55)	0.83	1.52
Regional	71.51^† ^ (35.45)	−15.09^† ^ (−6.70)	−9.02^† ^ (−4.09)	5.31^*♯*^ (2.30)	0.62^† ^ (36.64)	229.06^*♯*^ (2.53)	−199.79^*♯*^ (−2.34)	−183.03^*♯*^ (−2.21)	−150.44* (−1.90)	0.17^† ^ (4.21)	0.50^† ^ (4.69)			0.64	1.42
National	71.31^† ^ (26.12)	−10.56^† ^ (−3.72)	−5.62^*♯*^ (−2.01)	8.06^† ^ (2.98)	0.69^† ^ (36.18)	108.86^*♯*^ (2.53)	−91.94^† ^ (−2.24)	−84.06^*♯*^ (−2.12)	−62.42 (−1.58)	0.37^† ^ (3.96)	0.47^† ^ (5.42)			1.67	2.32

**Table:** (c) Northern China

API	c	S_2_	S_3_	S_4_	AR	ω	S_2_′	S_3_′	S_4_′	ARCH (*α*)	GARCH (*β*)	Θ_1_	Θ_2_	Q(5)	Q(10)
Beijing, regional, and national															

Beijing	104.17^†^ (19.62)	−20.64^†^ (−3.87)	−19.15^†^ (−3.56)	−15.07^†^ (−2.81)	0.51^†^ (22.21)	2026.18^†^ (19.97)	−1758.96^†^ (28.37)	−1461.45^†^ (16.29)	−1338.17^†^ (12.02)	0.40^†^ (4.41)	0.32^†^ (2.72)	0.02 (1.53)	0.88^†^ (9.12)	2.15	5.12
Regional	82.30^†^ (30.73)	−6.09^*♯*^ (−2.01)	−2.59 (−0.85)	12.62^†^ (3.78)	0.64^†^ (33.77)	64.33 (1.01)	−36.96 (−0.84)	−0.85 (−0.03)	5.85 (0.25)	0.17* (1.65)	0.77^†^ (5.34)			19.63^†^	21.2^*♯*^
National	79.44^†^ (35.24)	−6.51^*♯*^ (−2.51)	−3.41 (−1.34)	11.42^†^ (4.17)	0.67^†^ (37.50)	76.88* (1.78)	−51.53^†^ (−1.57)	−11.91 (−0.53)	4.69^†^ (0.20)	0.28^†^ (2.74)	0.63^†^ (4.54)			2.48	9.85

Huhehaote, regional, and national															

Huhehaote	80.95^†^ (13.80)	−27.22^†^ (−4.33)	−19.58^†^ (−2.95)	6.23 (0.96)	0.66^†^ (32.39)	898.32^†^ (4.03)	−864.09^†^ (−4.13)	−838.20^†^ (−4.15)	−737.24^†^ (−4.24)	0.28^†^ (3.53)	0.64^†^ (6.73)	0.02^†^ (2.70)	0.90^†^ (20.13)	2.62	3.99
Regional	93.68^†^ (26.43)	−9.93^†^ (−2.64)	−4.88 (−1.20)	8.51* (1.77)	0.68^†^ (40.40)	51.52 (0.54)	−41.16 (−0.51)	−14.73 (−0.25)	−3.80 (−0.09)	0.09 (1.41)	0.87^†^ (7.30)			67.54^†^	77.31^†^
National	84.42^†^ (36.09)	−10.99^†^ (−4.48)	−5.72^*♯*^ (−2.26)	7.81^†^ (2.91)	0.74^†^ (50.63)	117.55^*♯*^ (2.04)	−97.18* (−1.89)	−59.62 (−1.49)	−26.66 (−0.80)	0.28^†^ (4.85)	0.47^†^ (3.47)			4.37	45.35^†^

Shijiazhuang, regional, and national															

Shijiazhuang	96.16^†^ (26.51)	−9.98^†^ (−2.65)	−9.87^*♯*^ (−2.25)	7.81* (1.73)	0.70^†^ (38.97)	623.90^†^ (3.41)	−435.56^†^ (−2.60)	−235.27 (−1.18)	−113.38 (−0.78)	0.40^†^ (5.11)	0.29^†^ (3.24)	0.03^†^ (3.78)	0.77^†^ (14.77)	0.7	7.41
Regional	92.35^†^ (28.08)	−11.59^†^ (−3.28)	−5.65 (−1.52)	7.79* (1.89)	0.67^†^ (39.52)	78.43 (0.89)	−64.45 (−0.87)	−34.74 (−0.63)	−12.10 (−0.38)	0.11^*♯*^ (1.99)	0.84^†^ (7.93)			179.27^†^	189.53^†^
National	86.39^†^ (33.54)	−11.82^†^ (−4.47)	−5.79^*♯*^ (−2.12)	7.44^*♯*^ (2.53)	0.71^†^ (48.40)	173.03* (1.83)	−141.96* (−1.71)	−89.57 (−1.41)	−35.47 (−0.70)	0.28^†^ (4.59)	0.46^†^ (2.67)			9.74*	26.89^†^

Taiyuan, regional, and national															

Taiyuan	89.844^†^ (23.87)	−6.73* (−1.72)	2.23 (0.52)	12.92^†^ (2.71)	0.65^†^ (34.44)	*Z*	−46.76* (−1.67)	−25.40 (−0.96)	−13.77 (−0.53)	0.10^†^ (5.03)	0.88^†^ (35.53)	0.01 (1.15)	0.70^†^ (5.13)	10.93*	21.65^*♯*^
Regional	92.47^†^ (21.38)	−14.56^†^ (−3.29)	−11.21^*♯*^ (−2.43)	6.28 (1.17)	0.71^†^ (40.61)	96.69 (0.68)	−84.85 (−0.68)	−60.19 (−0.57)	−48.95 (−0.54)	0.11 (1.53)	0.84^†^ (6.23)			76.46^†^	82.57^†^
National	84.62^†^ (30.67)	−11.46^†^ (−4.07)	−7.41^*♯*^ (−2.51)	7.27^†^ (2.50)	0.74^†^ (45.13)	165.94^*♯*^ (2.53)	−136.82^*♯*^ (−2.29)	−87.56* (−1.67)	−56.59 (−1.19)	0.33^†^ (4.69)	0.40^†^ (3.22)			8.95	88.09^†^

Tianjin, regional, and national															

Tianjin	81.29^†^ (34.31)	−6.20^*♯*^ (−2.14)	−2.67 (−0.90)	11.63^†^ (3.70)	0.57^†^ (28.01)	63.60* (1.79)	−24.23 (−0.90)	12.06 (0.56)	9.22 (0.43)	0.16^*♯*^ (2.53)	0.79^†^ (10.37)	0.10^†^ (6.36)	0.37^†^ (3.42)	3.04	4.33
Regional	102.78^†^ (21.72)	−19.49^†^ (−4.07)	−16.91^†^ (−3.39)	−8.40* (−1.71)	0.54^†^ (24.46)	1831.75^†^ (20.78)	−1584.64^†^ (27.10)	−1259.84^†^ (−20.64)	−1170.60^†^ (−15.75)	0.41^†^ (5.11)	0.20* (1.77)			3.13	9.96
National	93.47^†^ (25.52)	−16.94^†^ (−4.56)	−13.50^†^ (−3.46)	−2.74 (−0.71)	0.57^†^ (27.47)	820.45^†^ (9.42)	−710.87^†^ (−8.93)	−566.76^†^ (−7.32)	−510.70^†^ (−7.25)	0.42^†^ (5.16)	0.23^*♯*^ (2.12)			4.54	5.02

**Table:** (d) Northeast

API	c	S_2_	S_3_	S_4_	AR	ω	S_2_′	S_3_′	S_4_′	ARCH (*α*)	GARCH (*β*)	Θ_1_	Θ_2_	Q(5)	Q(10)
Changchun, regional, and national															

Changchun	76.97^† ^ (45.63)	−17.53^† ^ (−9.38)	−9.38^† ^ (−4.48)	11.21^† ^ (5.12)	0.49^† ^ (23.20)	61.17 (1.10)	−51.96 (−1.06)	−34.32 (−0.78)	−22.65 (−0.62)	0.05^† ^ (2.63)	0.88* (16.81)	0.04^† ^ (6.94)	0.90^† ^ (57.24)	14.33^*♯*^	16.04
Regional	84.80^† ^ (23.00)	−14.69^† ^ (−3.88)	−8.38^*♯*^ (−2.15)	10.68^† ^ (2.74)	0.64^† ^ (36.89)	93.05* (1.68)	−84.49 (−1.64)	−69.25 (−1.47)	−36.79 (−0.94)	0.17^† ^ (3.93)	0.76^† ^ (10.86)			3.66	5.59
National	81.04^† ^ (32.15)	−12.63^† ^ (−4.78)	−6.95^*♯*^ (−2.56)	9.52^† ^ (3.08)	0.72^† ^ (46.24)	43.96* (1.84)	−40.10* (−1.80)	−31.02 (−1.55)	−14.92 (−0.86)	0.16^† ^ (4.18)	0.78^† ^ (12.91)			25.75^† ^	30.68^† ^

Dalian, regional, and national															

Dalian	70.41^† ^ (14.47)	−15.04^† ^ (−2.95)	−10.05^*♯*^ (−2.10)	1.93 (0.35)	0.54^† ^ (13.23)	555.30^*♯*^ (2.14)	−495.80^*♯*^ (−2.00)	−496.67^*♯*^ (−2.00)	−430.21* (−1.73)	0.35^*♯*^ (2.02)	0.50^† ^ (6.30)	0.02^† ^ (3.86)	0.96^† ^ (72.85)	0.99	1.53
Regional	88.39^† ^ (35.73)	−15.21^† ^ (−5.89)	−10.57^† ^ (−3.95)	9.06^† ^ (3.08)	0.60^† ^ (34.50)	74.50^*♯*^ (2.41)	−60.09^*♯*^ (−2.18)	−44.22* (−1.75)	−24.32 (−1.09)	0.14^† ^ (4.06)	0.78^† ^ (17.69)			3.19	5.33
National	74.27^† ^ (47.99)	−11.09^† ^ (−6.29)	−6.50^† ^ (−3.69)	6.08^† ^ (3.19)	0.71^† ^ (45.84)	30.93^*♯*^ (2.13)	−22.59^*♯*^ (−2.15)	−15.26^*♯*^ (−2.13)	−7.06 (−1.28)	0.23^† ^ (4.68)	0.66^† ^ (6.26)			4.85	9.1

Haerbin, regional, and national															

Haerbin	79.00^† ^ (15.31)	−13.51^*♯*^ (−2.46)	−4.56 (−0.82)	16.90^† ^ (3.25)	0.61^† ^ (24.86)	199.95 (1.44)	−193.80 (−1.44)	−180.38 (−1.43)	−95.15 (−0.95)	0.17^† ^ (3.59)	0.79^† ^ (13.40)	0.02^† ^ (5.60)	0.92^† ^ (65.94)	1.65	3.74
Regional	80.11^† ^ (47.21)	−17.34^† ^ (−9.22)	−10.33^† ^ (−5.02)	10.43^† ^ (4.80)	0.54^† ^ (27.96)	48.24 (1.51)	−41.36 (−1.45)	−27.12 (−1.04)	−19.19 (−0.84)	0.06^† ^ (2.62)	0.87^† ^ (20.60)			25.43^† ^	26.9^† ^
National	78.41^† ^ (46.13)	−15.35^† ^ (−8.26)	−9.00^† ^ (−4.92)	9.75^† ^ (4.75)	0.65^† ^ (36.64)	26.96 (1.53)	−23.74 (−1.48)	−15.36 (−1.07)	−10.02 (−0.79)	0.09^† ^ (2.67)	0.85^† ^ (17.57)			37.43^† ^	40.29^† ^

Shenyang, regional, and national															

Shenyang	89.73^† ^ (30.29)	−10.57^† ^ (−3.54)	−7.69^*♯*^ (−2.54)	10.05^† ^ (3.04)	0.56^† ^ (29.14)	86.81^*♯*^ (2.13)	−68.75* (−1.86)	−49.39 (−1.42)	−35.91 (−1.14)	0.15^† ^ (3.73)	0.81^† ^ (20.59)	0.03^† ^ (4.06)	0.92^† ^ (36.20)	1.82	3.63
Regional	76.66^† ^ (44.45)	−17.68^† ^ (−9.30)	−10.91^† ^ (−5.45)	7.28^† ^ (3.57)	0.57^† ^ (27.35)	53.10 (1.50)	−48.40 (−1.48)	−38.02 (−1.25)	−30.94 (−1.14)	0.09^† ^ (2.88)	0.85^† ^ (17.21)			4.06	6.59
National	74.05^† ^ (37.50)	−11.97^† ^ (−5.57)	−7.12^† ^ (−3.53)	9.11^† ^ (3.83)	0.72^† ^ (42.11)	26.61 (0.75)	−23.83 (−0.76)	−17.74 (−0.69)	−11.27 (−0.65)	0.18 (1.24)	0.77^† ^ (3.58)				

**Table:** (e) Northwest

API	c	S_2_	S_3_	S_4_	AR	ω	S_2_′	S_3_′	S_4_′	ARCH (*α*)	GARCH (*β*)	Θ_1_	Θ_2_	Q(5)	Q(10)
Lanzhou, regional, and national															

Lanzhou	117.69^† ^ (10.74)	−41.57^† ^ (−3.66)	−31.43^† ^ (−2.73)	8.62 (0.58)	0.65^† ^ (22.14)	582.16^† ^ (3.90)	−548.06^† ^ (−3.74)	−534.41^† ^ (−3.62)	−320.70 (−1.31)	0.22^† ^ (4.10)	0.79^† ^ (25.28)	0.02^† ^ (7.30)	0.95^† ^ (162.9)	1.22	2.64
Regional	81.58^† ^ (33.93)	−16.39^† ^ (−6.33)	−11.51^† ^ (−4.41)	15.91^† ^ (5.86)	0.64^† ^ (31.19)	36.73 (1.43)	−33.75 (−1.43)	−28.01 (−1.24)	−9.09 (−0.55)	0.11* (1.82)	0.86^† ^ (12.74)			23.39^† ^	25.27^† ^
National	80.16^† ^ (21.37)	−11.69^† ^ (−3.05)	−5.23 (−1.46)	9.52^† ^ (2.66)	0.75^† ^ (39.18)	70.81 (0.99)	−61.50 (−0.94)	−62.76 (−0.96)	−41.94 (−0.75)	0.16^† ^ (3.06)	0.80^† ^ (8.72)			0.53	0.8

Xian, regional, and national															

Xian	84.17^† ^ (14.44)	−5.20 (−0.90)	0.79 (0.14)	15.80^*♯*^ (2.57)	0.64^† ^ (29.90)	185.60 (1.18)	−158.11 (−1.11)	−138.12 (−1.05)	−115.80 (−0.94)	0.23^† ^ (3.43)	0.69^† ^ (5.71)	0.02^† ^ (6.60)	0.97^† ^ (191.6)	2.37	10.77
Regional	100.38^† ^ (18.09)	−33.10^† ^ (−5.80)	−25.96^† ^ (−4.52)	11.96* (1.81)	0.67^† ^ (32.61)	169.81^† ^ (2.61)	−162.12^*♯*^ (−2.56)	−151.89^*♯*^ (−2.45)	−89.60 (−1.50)	0.16^† ^ (4.46)	0.82^† ^ (25.69)			6.63	10.47
National	77.93^† ^ (31.43)	−10.85^† ^ (−4.17)	−4.81* (−1.84)	7.63^*♯*^ (2.44)	0.83^† ^ (68.17)	14.52 (1.47)	−12.72 (−1.44)	−9.23 (−1.20)	−3.54 (−0.68)	0.12^† ^ (3.54)	0.82^† ^ (12.74)			27.55^† ^	34.17^† ^

Yinchuan, regional, and national															

Yinchuan	80.19^† ^ (33.17)	−22.54^† ^ (−8.94)	−17.07^† ^ (−6.84)	11.41^† ^ (4.31)	0.56^† ^ (24.60)	67.07 (1.44)	−64.53 (−1.43)	−57.04 (−1.29)	−31.29 (−0.77)	0.06^† ^ (2.97)	0.91^† ^ (31.57)	0.02^† ^ (5.53)	0.96^† ^ (99.98)	9.06	9.93
Regional	107.87 (15.81)	−31.07^† ^ (−4.39)	−22.46^† ^ (−3.16)	13.76 (1.59)	0.69^† ^ (27.01)	249.72^† ^ (2.67)	−232.40^† ^ (−2.60)	−224.93^*♯*^ (−2.59)	−115.87 (−1.43)	0.19^† ^ (4.62)	0.81^† ^ (20.29)			1.17	2.71
National	86.09^† ^ (22.68)	−15.53^† ^ (−4.08)	−10.03^† ^ (−2.63)	9.43^*♯*^ (2.14)	0.80^† ^ (56.83)	58.08* (1.83)	−53.48* (−1.77)	−47.53* (−1.68)	−26.11 (−1.15)	0.16^† ^ (5.48)	0.78^† ^ (14.84)			3.4	5.51

Wulumuqi, regional, and national															

Wulumuqi	72.12^† ^ (10.04)	−18.49^† ^ (−2.79)	−3.91 (−0.53)	78.21^† ^ (6.58)	0.69^† ^ (31.18)	377.67 (1.19)	−356.11 (−1.17)	−306.93 (−1.13)	798.85 (1.07)	0.35^† ^ (3.61)	0.64^† ^ (4.97)	0.01^† ^ (3.86)	0.96^† ^ (76.15)	1.2	2.08
Regional	97.52^† ^ (20.62)	−26.81^† ^ (−5.57)	−21.36^† ^ (−4.36)	10.93^*♯*^ (1.96)	0.70^† ^ (37.94)	114.08^*♯*^ (2.18)	−108.68^*♯*^ (−2.14)	−100.45^*♯*^ (−2.03)	−60.11 (−1.35)	0.16^† ^ (4.74)	0.82^† ^ (24.23)			5.61	8.88
National	74.48^† ^ (31.22)	−10.91^† ^ (−4.40)	−5.90^*♯*^ (−2.45)	6.95^*♯*^ (2.48)	0.83^† ^ (60.64)	23.98 (1.54)	−21.50 (−1.50)	−17.37 (−1.38)	−9.29 (−0.99)	0.15^† ^ (3.86)	0.74^† ^ (8.11)			11.26^*♯*^	14.45

**Table:** (f) Qinghai-Tibet

API	c	S_2_	S_3_	S_4_	AR	ω	S_2_′	S_3_′	S_4_′	ARCH (*α*)	GARCH (*β*)	Θ_1_	Θ_2_	Q(5)	Q(10)
Lasa, regional, and national															

Lasa	57.46^† ^ (36.51)	−19.33^† ^ (−9.88)	−10.98^† ^ (−5.77)	0.64 (0.28)	0.62^† ^ (37.45)	17.00^*♯*^ (2.14)	−10.86* (−1.87)	−7.40 (−1.48)	22.71^*♯*^ (2.45)	0.08^† ^ (3.50)	0.86^† ^ (21.76)	0.01^† ^ (3.62)	0.97^† ^ (96.27)	23.74^† ^	25.21^† ^
Regional	88.46^† ^ (12.35)	−19.68^† ^ (−3.21)	−11.83* (−1.70)	1.54^*♯*^ (0.22)	0.60^† ^ (18.41)	715.67^*♯*^ (2.57)	−676.97^† ^ (−2.60)	−671.37^†^ ^ ^ (−2.67)	−538.08^*♯*^ (−2.45)	0.51 (1.32)	0.58^† ^ (2.94)			1.03	1.34
National	73.37^† ^ (32.40)	−9.32^† ^ (−3.87)	−3.94* (−1.69)	8.97^† ^ (3.04)	0.85^† ^ (60.87)	13.92 (1.46)	−11.98 (−1.40)	−9.11 (−1.23)	−0.31 (−0.08)	0.12^† ^ (3.70)	0.79^† ^ (9.98)			14.38^*♯*^	18.1^*♯*^

Xining, regional, and national															

Xining	88.46^† ^ (12.35)	−19.68^† ^ (−3.22)	−11.83* (−1.70)	1.54 (0.22)	0.60^† ^ (18.41)	715.67^*♯*^ (2.57)	−676.97^† ^ (−2.60)	−671.37^† ^ (−2.67)	−538.08^*♯*^ (−2.45)	0.51 (1.32)	0.58^† ^ (2.94)	0.02^† ^ (5.62)	0.96^† ^ (154.9)	0.84	1.56
Regional	57.46^† ^ (36.51)	−19.33^† ^ (−9.88)	−10.98^† ^ (−5.77)	0.64 (0.28)	0.62^† ^ (37.45)	17.00^*♯*^ (2.14)	−10.86* (−1.87)	−7.40 (−1.48)	22.71^*♯*^ (2.45)	0.08^† ^ (3.50)	0.86^† ^ (21.76)			23.49^† ^	24.88^† ^
National	95.83^† ^ (16.96)	−26.73^† ^ (−4.65)	−18.82^† ^ (−3.24)	10.41 (1.50)	0.72^† ^ (33.53)	150.38^*♯*^ (2.55)	−143.90^*♯*^ (−2.49)	−138.06^*♯*^ (−2.44)	−100.98* (−1.88)	0.21^† ^ (4.56)	0.80^† ^ (23.41)			2.86	4.75

**Table:** (g) Southern China

API	c	S_2_	S_3_	S_4_	AR	ω	S_2_′	S_3_′	S_4_′	ARCH (*α*)	GARCH (*β*)	Θ_1_	Θ_2_	Q(5)	Q(10)
Guangzhou, regional, and national															

Guangzhou	63.87^† ^ (32.45)	−6.48^† ^ (−2.63)	1.52 (0.55)	8.02^† ^ (2.89)	0.69^† ^ (45.66)	221.58^*♯*^ (2.25)	−131.67^*♯*^ (−2.23)	−72.52* (−1.90)	69.64* (1.76)	0.21^† ^ (3.67)	0.24 (0.88)	0.05^† ^ (4.29)	0.57^† ^ (3.85)	8.8	23.22^† ^
Regional	46.88^† ^ (43.80)	−10.48^† ^ (−7.92)	5.58^† ^ (3.66)	10.15^† ^ (6.65)	0.70^† ^ (50.57)	42.45^† ^ (2.61)	−14.80^*♯*^ (−2.14)	−3.69 (−0.80)	13.99^*♯*^ (2.06)	0.14^† ^ (4.30)	0.44^*♯*^ (2.44)			3.23	10.26
National	49.64^† ^ (45.92)	−10.31^† ^ (−7.71)	4.62^† ^ (3.03)	9.39^† ^ (6.15)	0.73^† ^ (54.91)	30.85^*♯*^ (2.53)	−11.55^*♯*^ (−2.09)	−3.51 (−1.03)	10.89^*♯*^ (2.15)	0.14^† ^ (3.95)	0.49^† ^ (2.92)			3.88	6.07

Shenzhen, regional, and national															

Shenzhen	51.54^† ^ (38.89)	−10.18^† ^ (−5.87)	8.41^† ^ (4.53)	10.77^† ^ (5.31)	0.66^† ^ (45.27)	20.55 (0.99)	−4.70 (−0.62)	−0.63* (−0.19)	13.20 (1.38)	0.06* (1.79)	0.83^† ^ (6.25)	0.01^† ^ (4.86)	0.99^† ^ (350.2)	15.56^† ^	18.27*
Regional	48.53^† ^ (41.70)	−10.65^† ^ (−7.67)	2.42 (1.54)	9.66^† ^ (5.99)	0.71^† ^ (53.73)	61.26^† ^ (4.64)	−25.42^† ^ (−3.37)	−10.85* (−1.63)	6.91 (1.02)	0.19^† ^ (6.17)	0.24* (1.94)			6.37	23.90^† ^
National	49.99^† ^ (43.19)	−10.66^† ^ (−7.75)	2.03 (1.31)	9.17^† ^ (5.74)	0.73^† ^ (55.81)	51.39^† ^ (4.58)	−22.36^† ^ (−3.46)	−10.27* (−1.83)	6.12 (1.05)	0.19^† ^ (6.01)	0.28^*♯*^ (2.24)			6.55	31.65^† ^

Zhuhai, regional, and national															

Zhuhai	43.34^† ^ (32.95)	−12.58^† ^ (−7.80)	2.33 (1.27)	10.36^† ^ (5.93)	0.70^† ^ (48.40)	3.78 (1.01)	−0.67 (−0.40)	−0.02 (−0.02)	1.26 (1.13)	0.06^*♯*^ (2.39)	0.91^† ^ (18.07)	0.01^† ^ (4.72)	0.99^† ^ (454.5)	27.76^† ^	32.70^† ^
Regional	54.27^† ^ (40.20)	−8.81^† ^ (−5.31)	6.72^† ^ (3.65)	10.03^† ^ (5.17)	0.70^† ^ (52.46)	95.13^*♯*^ (2.53)	−36.07^*♯*^ (−2.36)	−19.57* (−1.73)	38.50^*♯*^ (2.15)	0.13^† ^ (4.68)	0.33 (1.48)			3.23	19.45^*♯*^
National	55.12^† ^ (41.17)	−8.92^† ^ (−5.43)	6.23^† ^ (3.42)	9.72^† ^ (5.11)	0.71^† ^ (54.64)	80.44^*♯*^ (2.37)	−31.54^*♯*^ (−2.23)	−17.74* (−1.74)	32.49^*♯*^ (2.10)	0.12^† ^ (4.41)	0.37* (1.62)			7.26	10.18

Zhanjiang, regional, and national															

Zhanjiang	46.59^† ^ (75.39)	−0.66 (0.80)	0.66 (0.83)	1.27* (1.67)	0.82^† ^ (71.11)	0.05 (1.14)	0.07 (1.05)	−0.02 (0.32)	0.07 (1.04)	0.04^† ^ (6.14)	0.95^† ^ (127.6)	0.06^† ^ (4.16)	0.69^† ^ (5.70)	12.08^*♯*^	18.65^*♯*^
Regional	39.49^† ^ (44.27)	−7.28^† ^ (−6.59)	5.11^† ^ (3.71)	8.71^† ^ (7.24)	0.76^† ^ (51.02)	4.07 (1.28)	−1.72 (−1.04)	0.25 (0.38)	1.16 (1.40)	0.09^† ^ (2.69)	0.82^† ^ (8.76)			8.1	20.89^*♯*^
National	46.43^† ^ (43.69)	−6.23^† ^ (−4.45)	4.84^† ^ (3.29)	7.53^† ^ (5.55)	0.83^† ^ (61.97)	12.16^† ^ (3.46)	−6.61^† ^ (−2.95)	−0.33 (−0.27)	3.20^*♯*^ (1.99)	0.15^† ^ (4.85)	0.54^† ^ (5.11)			26.87	94.48^† ^

Haikou, regional, and national															

Haikou	34.09^† ^ (38.43)	−7.02^† ^ (−5.93)	5.81^† ^ (3.85)	8.10^† ^ (6.71)	0.73^† ^ (45.76)	2.20 (0.97)	−0.33 (0.35)	1.04 (1.17)	1.24 (1.40)	0.07^*♯*^ (2.38)	0.89^† ^ (13.55)	0.01^† ^ (5.54)	0.98^† ^ (189.8)	2.61	6.26
Regional	47.98^† ^ (70.93)	−2.29^† ^ (−2.67)	1.98^*♯*^ (2.25)	3.80^† ^ (4.26)	0.79^† ^ (67.57)	0.03 (0.49)	0.14^*♯*^ (2.14)	0.09 (1.41)	0.29^† ^ (2.97)	0.03^† ^ (5.69)	0.96^† ^ (142.8)			13.26*	15.63
National	51.68^† ^ (54.92)	−2.49^*♯*^ (−2.02)	2.14* (1.80)	4.84^† ^ (4.24)	0.85^† ^ (73.05)	−0.01 (−0.19)	0.12^*♯*^ (2.11)	0.20^† ^ (3.87)	0.43^† ^ (4.66)	0.03^† ^ (4.60)	0.96^† ^ (124.2)			39.99^† ^	40.52^† ^

**Table:** (h) Southeast

API	c	S_2_	S_3_	S_4_	AR	ω	S_2_′	S_3_′	S_4_′	ARCH (*α*)	GARCH (*β*)	Θ_1_	Θ_2_	Q(5)	Q(10)
Fuzhou, regional, and national															

Fuzhou	65.16^† ^ (43.51)	−13.04^† ^ (−7.14)	−11.25^† ^ (−6.18)	−3.81* (−1.86)	0.58^† ^ (36.60)	78.15 (1.38)	−45.11 (−1.37)	−38.56 (−1.29)	13.39 (1.40)	0.09^*♯*^ (2.04)	0.70^† ^ (3.83)	0.03^† ^ (7.89)	0.95^† ^ (146.7)	17.67^*♯*^	22.24^†^
Regional	57.29^† ^ (37.40)	−15.79^† ^ (−7.67)	−1.93 (−1.05)	3.46* (1.78)	0.66^† ^ (46.08)	24.65^† ^ (3.18)	−13.49^† ^ (−3.11)	−12.14^† ^ (−2.83)	−1.78 (−0.58)	0.03* (1.86)	0.84^† ^ (19.40)				
National	62.54^† ^ (37.39)	−10.29^† ^ (−5.02)	−2.32 (−1.20)	4.34^*♯*^ (2.20)	0.80^† ^ (58.50)	8.14 (0.84)	−5.72 (−0.84)	−3.19 (−0.69)	1.79 (1.55)	0.05 (1.15)	0.85^† ^ (5.59)				

Shantou, regional, and national															

Shantou	50.82^† ^ (34.11)	−12.34^† ^ (−6.29)	4.46^*♯*^ (2.42)	8.93^† ^ (4.70)	0.70^† ^ (48.90)	24.24^† ^ (2.83)	−11.62^*♯*^ (−2.40)	−11.74^*♯*^ (−2.44)	−3.85 (−1.07)	0.09^† ^ (4.33)	0.78^† ^ (13.97)	0.02^† ^ (6.38)	0.96^† ^ (151.9)	21.14^† ^	24.22^† ^
Regional	60.34^† ^ (38.90)	−16.38^† ^ (−8.30)	−5.07^† ^ (−2.75)	0.90 (0.45)	0.64^† ^ (43.43)	26.25^† ^ (3.31)	−15.74^† ^ (−3.14)	−11.98^† ^ (−2.66)	0.26 (0.08)	0.02 (1.21)	0.88^† ^ (22.26)			10.99^*♯*^	13.08
National	59.00^† ^ (35.81)	−9.24^† ^ (−3.83)	−0.24 (−0.13)	6.19^† ^ (3.10)	0.79^† ^ (60.31)	3.95 (0.51)	−2.47 (−0.48)	−1.27 (−0.31)	2.21^† ^ (3.08)	0.02 (0.67)	0.93^† ^ (8.49)			27.01^† ^	35.09^† ^

Xiamen, regional, and national															

Xiamen	59.30^† ^ (38.55)	−17.24^† ^ (−8.37)	−3.68^*♯*^ (−1.98)	1.86 (0.94)	0.63^† ^ (42.92)	32.43^† ^ (3.02)	−17.00^† ^ (−3.14)	−15.24^† ^ (−2.82)	−3.10 (−0.83)	0.03* (1.64)	0.86^† ^ (16.32)	0.02^† ^ (6.64)	0.96^† ^ (125.0)	14.65^*♯*^	15.4
Regional	56.74^† ^ (39.19)	−12.66^† ^ (−7.28)	−2.45 (−1.42)	3.48* (1.82)	0.67^† ^ (46.99)	31.21^† ^ (2.72)	−22.11^† ^ (−2.66)	−16.77^*♯*^ (−2.46)	2.80 (0.71)	0.08^† ^ (2.93)	0.76^† ^ (10.10)			15.95^*♯*^	20.42^*♯*^
National	58.83^† ^ (35.93)	−8.93^† ^ (−4.50)	−0.45 (−0.24)	5.60^† ^ (2.89)	0.79^† ^ (58.44)	14.82* (1.82)	−10.77* (−1.77)	−7.17* (−1.65)	3.70* (1.73)	0.09^† ^ (2.60)	0.74^† ^ (6.34)			14.28^*♯*^	17.92^*♯*^

**Table:** (i) Southwest

API	c	S_2_	S_3_	S_4_	AR	ω	S_2_′	S_3_′	S_4_′	ARCH (*α*)	GARCH (*β*)	Θ_1_	Θ_2_	Q(5)	Q(10)
Chengdu, regional, and national															

Chengdu	86.59^† ^ (23.71)	−14.89^† ^ (−3.87)	−10.27^*♯*^ (−2.57)	4.53 (1.07)	0.62^† ^ (31.66)	112.81 (1.27)	−78.13 (−1.06)	−68.71 (−0.97)	−43.38 (−0.67)	0.19^† ^ (3.67)	0.70^† ^ (6.22)	0.03^† ^ (5.29)	0.90^† ^ (32.86)	0.35	0.61
Regional	90.71^† ^ (40.09)	−12.34^† ^ (−4.66)	−5.39* (−1.95)	5.24* (1.83)	0.73^† ^ (56.18)	47.58^*♯*^ (2.07)	−27.29^*♯*^ (−2.16)	−17.51* (−1.64)	−7.11 (−0.92)	0.08^*♯*^ (2.28)	0.79^† ^ (8.80)				
National	76.43^† ^ (40.92)	−9.45^† ^ (−4.53)	−3.64* (−1.65)	6.21^*♯*^ (2.52)	0.81^† ^ (67.32)	13.87^*♯*^ (2.21)	−9.14^*♯*^ (−2.18)	−4.66 (−1.49)	0.54 (0.23)	0.11^† ^ (3.21)	0.79^† ^ (10.92)				

Chongqing, regional, and national															

Chongqing	87.31^† ^ (31.93)	−12.52^† ^ (−4.05)	−6.41* (−1.93)	6.73* (1.94)	0.71^† ^ (52.51)	72.30^*♯*^ (2.47)	−44.52^*♯*^ (−2.40)	−28.50* (−1.77)	−8.19 (−0.61)	0.10^† ^ (3.04)	0.79^† ^ (11.39)	0.03^† ^ (4.42)	0.91^† ^ (31.38)	3.87	4.23
Regional	77.47^† ^ (32.55)	−11.85^† ^ (−4.63)	−5.66^*♯*^ (−2.10)	5.04* (1.82)	0.68^† ^ (48.72)	50.64* (1.89)	−31.83 (−1.59)	−25.68 (−1.36)	−11.87 (−0.70)	0.12^† ^ (3.24)	0.73^† ^ (7.50)			7.4	7.57
National	75.48^† ^ (40.47)	−10.34^† ^ (−5.14)	−2.62 (−1.24)	8.20^† ^ (3.53)	0.80^† ^ (62.93)	50.84^*♯*^ (2.09)	−38.88* (−1.89)	−28.93* (−1.63)	−9.92 (−0.75)	0.13^† ^ (3.66)	0.55^† ^ (4.11)			33.13^† ^	40.87^† ^

Guiyang, regional, and national															

Guiyang	68.18^† ^ (40.66)	−8.83^† ^ (−4.34)	−0.47 (−0.22)	5.65^*♯*^ (2.44)	0.62^† ^ (41.56)	9.71 (1.22)	−4.51 (−1.31)	1.27 (0.51)	0.99 (0.51)	0.04^*♯*^ (2.05)	0.93^† ^ (24.63)	0.02^† ^ (5.47)	0.95^† ^ (83.27)	76.21^† ^	80.31^† ^
Regional	75.56^† ^ (41.25)	−10.49^† ^ (−4.86)	−3.61* (−1.62)	5.39^*♯*^ (2.41)	0.76^† ^ (59.16)	29.96^*♯*^ (2.58)	−17.03^*♯*^ (−2.48)	−13.00^*♯*^ (−2.06)	−2.45 (−0.49)	0.08^† ^ (2.75)	0.77^† ^ (9.52)			2.83	4.7
National	70.32^† ^ (40.82)	−8.31^† ^ (−4.26)	−2.08 (−1.04)	5.67^† ^ (2.68)	0.84^† ^ (71.19)	10.98^*♯*^ (2.47)	−7.71^*♯*^ (−2.33)	−4.75* (−1.77)	0.97 (0.48)	0.10^† ^ (3.44)	0.77^† ^ (11.32)			29.04^† ^	30.74^† ^

Kunming, regional, and national															

Kunming	64.92^† ^ (65.84)	−8.37^† ^ (−6.43)	−2.31* (−1.66)	2.66* (1.89)	0.58^† ^ (34.43)	55.43^*♯*^ (2.26)	−6.54 (−1.00)	10.41 (1.42)	12.28 (1.47)	0.15^† ^ (3.40)	0.49^*♯*^ (2.56)	0.02^† ^ (4.49)	0.93^† ^ (53.81)	5.57	12.19
Regional	73.45^† ^ (39.39)	−10.19^† ^ (−4.71)	−1.88 (−0.84)	6.49^† ^ (2.72)	0.75^† ^ (61.52)	15.96 (0.94)	−9.37 (−1.01)	−5.37 (−0.72)	−1.07 (−0.36)	0.03 (1.07)	0.89^† ^ (8.33)			29.57^† ^	33.77^† ^
National	69.07^† ^ (37.55)	−8.49^† ^ (−3.87)	−2.09 (−1.00)	6.53^† ^ (2.67)	0.84^† ^ (67.95)	2.98 (0.29)	−2.18 (−0.29)	−0.53 (−0.10)	1.13 (1.26)	0.04 (0.52)	0.92^† ^ (5.03)			83.07^† ^	85.72^† ^

Nanning, regional, and national															

Nanning	52.73^† ^ (32.11)	−8.43^† ^ (−3.90)	8.77^† ^ (3.75)	10.03^† ^ (4.28)	0.72^† ^ (54.04)	79.25^† ^ (2.85)	−32.20^† ^ (−2.67)	3.39 (0.43)	40.26^*♯*^ (2.29)	0.08^† ^ (3.23)	0.48^† ^ (2.80)	0.01^*♯*^ (2.34)	0.93^† ^ (22.54)	7.02	8.16
Regional	73.66^† ^ (48.49)	−9.97^† ^ (−5.54)	−3.57* (−1.93)	4.50^*♯*^ (2.22)	0.70^† ^ (54.01)	4.93 (1.38)	−3.09* (−1.66)	−0.25 (−0.16)	−0.41 (0.46)	0.02^*♯*^ (1.99)	0.95^† ^ (34.76)			27.6^† ^	30.99^† ^
National	55.36^† ^ (42.36)	−5.06^† ^ (−3.08)	0.87 (0.57)	5.55^† ^ (3.46)	0.87^† ^ (77.70)	0.47 (0.62)	−0.27 (0.51)	0.31 (0.91)	0.82^† ^ (4.53)	0.03 (1.43)	0.95^† ^ (25.23)			17.84^† ^	20.62^*♯*^

Notes: This table reports the estimation results of DCC-GARCH models for the city APIs against regional and national APIs. Absolute t-ratios are indicated in parentheses. The Q(5) and *Q*(10) are, respectively, the Ljung-Box autocorrelations test (1978) of five and lags in the standardized squared residuals from the regression. ^†^ , ^*♯*^,* denote statistical significance at 1%, 5%, and 10% levels, respectively.

**Table 3:** Diagnostic tests.

	Without seasonality dummy			With seasonality dummy		
	Log(L)	AIC	BC	Log(L)	AIC	BC
(A) Central China						

Changsha, regional, and national	−40025.2	22.50	22.53	−39361.9	22.14	22.20
Hefei, regional, and national	−41104.9	23.10	23.14	−40345.2	22.69	22.76
Nanchang, regional, and national	−39886.3	22.42	22.45	−39360.6	22.13	22.20
Wuhan, regional, and national	−39781.4	22.36	22.40	−38801.2	21.82	21.89
Zhenzhou, regional, and national	−42877.7	24.10	24.13	−42039.1	23.64	23.71

(B) East China						

Hangzhou, regional, andnational	−37091.9	20.85	20.88	−36269.1	20.40	20.47
Jinan, regional, and national	−43761.2	24.60	24.63	−42476.7	23.89	23.95
Nanjing, regional, and national	−41336.6	23.23	23.27	−40413.4	22.73	22.79
Nantong, regional, and national	−34871.4	19.60	19.64	−33939.7	19.09	19.16
Qingdao, regional, and national	−39897.2	22.43	22.46	−38419	21.61	21.67
Shanghai, regional, and national	−35204.1	19.79	19.82	−34261.7	19.27	19.34
Suzhou, regional, and national	−33960.4	19.09	19.12	−34161.5	19.21	19.28
Wenzhou, regional, and national	−38456	21.62	21.65	−37964.4	21.35	21.42
Yantai, regional, and national	−41609.3	23.39	23.42	−39981.2	22.48	22.55

(C) North China						

Beijing, regional, and national	−42467.6	23.87	23.90	−41407.5	23.28	23.35
Huhehaote, regional, and national	−43935.3	24.69	24.73	−42821.3	24.08	24.15
Shijiazhuang, regional, and national	−43343.2	24.36	24.40	−42522.7	23.91	23.98
Taiyuan, regional, and national	−43172.6	24.27	24.30	−42804.1	24.07	24.14
Tianjin, regional, and national	−43504.4	24.45	24.49	−42123.2	23.69	23.75

(D) Northeast						

Changchun, regional, and national	−40528.1	22.78	22.81	−38723.9	21.78	21.84
Dalian, regional, and national	−44462.8	24.99	25.03	−42930.7	24.14	24.21
Haerbin, regional, and national	−41494.3	23.32	23.36	−39461.6	22.19	22.26
Shenyang, regional, and national	−42282.4	23.77	23.80	−40886.8	22.99	23.06

(E) Northwest						

Lanzhou, regional, and national	−48028.2	26.99	27.03	−45529.5	25.60	25.67
Xian, regional, and national	−45313.9	25.47	25.50	−43805.4	24.63	24.70
Yinchuan, regional, and national	−45947	25.82	25.86	−43833.9	24.65	24.72
Wulumuqi, regional, and national	−46248.1	25.99	26.03	−44306.9	24.91	24.98

(F) Qinghai-Tibet						

Lasa, regional, and national	−43834.8	24.64	24.67	−42628.7	23.97	24.04
Xining, regional, and national	−48225.4	27.10	27.14	−46366.3	26.07	26.14

(G) South China						

Guangzhou, regional, and national	−33688.9	18.94	18.97	−33282.6	18.72	18.79
Shenzhen, regional, and national	−31382.8	17.64	17.68	−30890.8	17.38	17.44
Zhuhai, regional, and national	−31246.1	17.57	17.60	−30971.2	17.42	17.49
Zhanjiang, regional, and national	−30018.4	16.88	16.91	−29767.6	16.75	16.81
Haikou, regional, and national	−30034.8	16.88	16.92	−29682.4	16.70	16.77

(H) Southeast						

Fuzhou, regional, and national	−39521.7	22.21	22.25	−39112.6	22.00	22.06
Shantou, regional, and national	−37615.6	21.14	21.18	−37231.3	20.94	21.01
Xiamen, regional, and national	−36573	20.56	20.59	−36170.9	20.34	20.41

(I) Southwest						

Chengdu, regional, and national	−40856.3	22.96	23.00	−40074.7	22.54	22.60
Chongqing, regional, and national	−40428.6	22.72	22.76	−39840.6	22.40	22.47
Guiyang, regional, and national	−38606.5	21.70	21.74	−38228.3	21.50	21.57
Kunming, regional, and national	−37677.7	21.18	21.21	−37317.2	20.99	21.05
Nanning, regional, and national	−37909.9	21.31	21.34	−37633.2	21.16	21.23

Notes: This table exhibits the comparison between DCC estimation without and with seasonality dummies. log(L) denotes Log-likelihood; AIC and BC denote Akaike and Schwarz information criteria, respectively.

**Table 4:** Seasonality significance.

API	Mean			Variance				Mean			Variance				Mean			Variance		
	equation			equation				equation			equation				equation			equation		
	S_2_	S_3_	S_4_	*S*_2_′	*S*_3_′	*S*_4_′	API	S_2_	S_3_	S_4_	*S*_2_′	*S*_3_′	*S*_4_′	API	S_2_	S_3_	S_4_	*S*_2_′	*S*_3_′	*S*_4_′
(A) Central China							(C) North China							(F) Qinghai-Tibet						
Changsha, regional, and national							Beijing, regional, and national							Xining, regional, and national						
Changsha	−			−			Beijing	−	−	−	−	−	−	Xining	−	−		−	−	−
Regional	−			−			Regional	−						Regional	−	−		−		+
National	−		+	−			National	−		+	−			National	−	−		−	−	−
Hefei, regional, and national							Huhehaote, regional, and national							(G) South China						
Hefei			−			−	Huhehaote	−	−		−	−	−	Guangzhou, regional, and national						
Regional			−			−	Regional	−		+				Guangzhou	−		+	−	−	+
National	+		−		−	−	National	−	−	+	−			Regional	−	+	+	−		+
Nanchang, regional, and national							Shijiazhuang, regional, and national							National	−	+	+	−		+
Nanchang	−			−			Shijiazhuang	−	−	+	−			Shenzhen, regional, and national						
Regional	−			−			Regional	−		+				Shenzhen	−	+	+			
National	−		+	−			National	−	−	+	−			Regional	−		+	−	−	
Wuhan, regional, and national							Taiyuan, regional, and national							National	−		+	−	−	
Wuhan	−		+				Taiyuan	−		+	−			Zhuhai, regional, and national						
Regional	−			−	−		Regional	−	−					Zhuhai	−		+			
National	−		+	−	−		National	−	−	+	−	−		Regional	−	+	+	−	−	+
Zhenzhou, regional, and national							Tianjin, regional, and national							National	−	+	+	−	−	+
Zhenzhou	−	−	+				Tianjin	−		+				Zhanjiang, regional, and national						
Regional	−			−	−		Regional	−	−	−	−	−	−	Zhanjiang			+			
National	−		+	−	−		National	−	−	−	−	−	−	Regional	−	+	+			
(B) East China							(D) Northeast							National	−	+	+	−		+
Hangzhou, regional, and national							Changchun, regional, and national							Haikou, regional, and national						
Hangzhou	−	−		−			Changchun	−	−	+				Haikou	−	+	+	−		
Regional	−	−		−			Regional	−	−	+				Regional	−	+	+	+		+
National	−	−		−	−		National	−	−	+	−			National	−	+	+	+	+	+
Jinan, regional, and national							Dalian, regional, and national							(H) Southeast						
Jinan	−	−		−	−		Dalian	−	−		−	−	−	Fuzhou, regional, and national						
Regional	−	−		−	−	−	Regional	−	−	+	−	−		Fuzhou	−	−	−			
National	−	−	+	−	−		National	−	−	+	−	−		Regional	−		+	−	−	
Nanjing, regional, and national							Haerbin, regional, and national							National	−		+			
Nanjing	−	−		−			Haerbin	−		+				Shantou, regional, and national						
Regional	−	−		−	−		Regional	−	−	+				Shantou	−	+	+	−	−	
National	−	−		−	−		National	−	−	+				Regional	−	−		−	−	
Nantong, regional, and national							Shenyang, regional, and national							National	−		+			+
Nantong	−			−	−		Shenyang	−	−	+	−			Xiamen, regional, and national						
Regional	−	−		−			Regional	−	−	+				Xiamen	−	−		−	−	
National	−	−		−			National	−	−	+				Regional	−		+	−	−	
Qingdao, regional, and national							(E) Northwest							National	−		+	−	−	+
Qingdao	−	−	+	−	−	−	Lanzhou, regional, and national							(I) Southwest						
Regional	−	−		−	−		Lanzhou	−	−	+	−	−		Chengdu, regional, and national						
National	−	−	+	−	−	−	Regional	−	−	+				Chengdu	−	−				
Shanghai, regional, and national							National	−		+				Regional	−	−	+	−	−	
Shanghai				−	−	−	Xian, regional, and national							National	−	−	+	−		
Regional	−	−		−	−		Xian			+				Chongqing, regional, and national						
National	−	−		−			Regional	−	−	+	−	−		Chongqing	−	−	+	−	−	
Suzhou, regional, and national							National	−	−	+				Regional	−	−	+			
Suzhou	−	−		−			Yinchuan, regional, and national							National	−		+	−	−	
Regional	−	−					Yinchuan	−	−	+				Guiyang, regional, and national						
National	−			−	−		Regional	−	−		−	−		Guiyang	−		+			
Wenzhou, regional, and national							National	−	−	+	−	−		Regional	−	−	+	−	−	
Wenzhou	−	−	−	−	−		Wulumuqi, regional, and national							National	−		+	−	−	
Regional	−	−		−			Wulumuqi	−		+				Kunming, regional, and national						
National	−	−					Regional	−	−	+	−	−		Kunming	−	−	+			
Yantai, regional, and national							National	−	−	+				Regional	−		+			
Yantai	−	−		−	−	−	Panel F: Qinghai-Tibet							National	−		+			
Regional	−	−	+	−	−	−	Lasa, regional, and national							Nanning, regional, and national						
National	−	−	+	−	−		Lasa	−	−		−		+	Nanning	−	+	+	−		+
							Regional	−	−	+	−	−	−	Regional	−	−	+	−		
							National	−	−	+				National	−		+			+

Notes: S_2_, S_3_, S_4_ and *S*_2_′, *S*_3_′, *S*_4_′ denote summer, autumn, and winter, respectively. −, + denote the negative and positive signs of the coefficients of seasonality dummies which were significant at 5% level. The empty entries mean that the corresponding coefficients are not significant at 5% level.
